# Supplementary material for: Bidirectional pilus processing in the Tad pilus system motor CpaF
Source: Nat Commun. 2024 Aug 5;15:6635. doi: 10.1038/s41467-024-50280-6 (PMC11300603; doi:10.1038/s41467-024-50280-6)
Supplement: Supplementary file 1 — Supplementary Information [file 41467_2024_50280_MOESM1_ESM.pdf]

Bidirectional pilus processing in the Tad pilus system motor CpaF

Michael Hohl[1], Emma J Banks[2], Max P Manley[1], Tung BK Le[2] and Harry H Low*[1]

[1]Department of Infectious Disease, Imperial College, London, SW7 2AZ, UK

[2]Department of Molecular Microbiology, John Innes Centre, Norwich, NR4 7UH, UK

*Corresponding author:

Harry H Low

Department of Infectious Disease, Imperial College, London, SW7 2AZ, UK

Email: h.low@imperial.ac.uk

Supplementary Figures

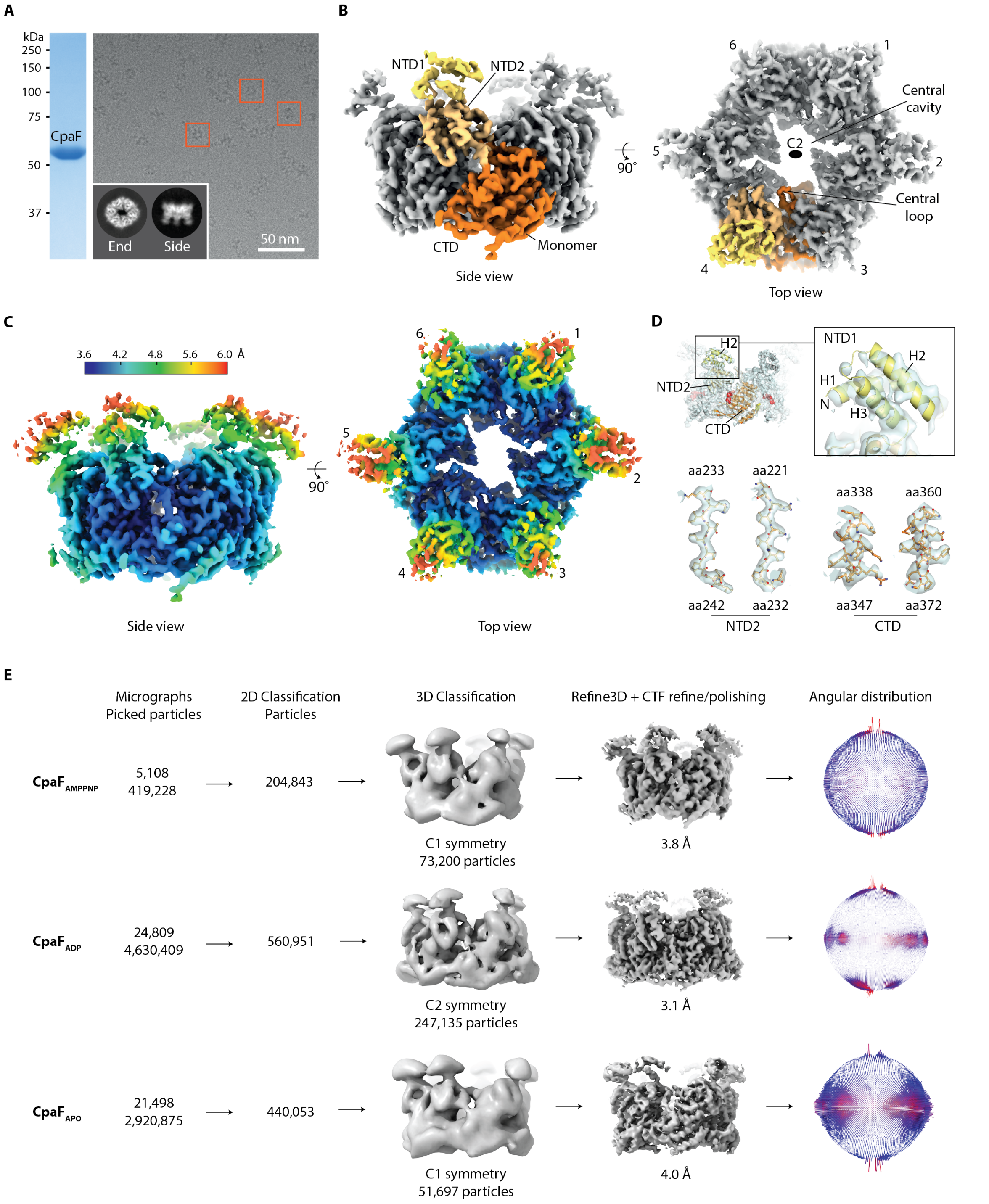

Supplementary Figure 1. CpaF$_{AMPPNP}$ cryo-EM map, model fit and processing. A, Purification and vitrification of CpaF incubated with AMPPNP. (Left) SDS-PAGE analysis of purified CpaF with theoretical mass of 52 kDa including residual C-terminal residues ALEVLFQ after tag cleavage. (Right) Typical cryo-EM micrograph with associated class averages (inset). Red box shows example boxed particles. **B**, Side and top views of CpaF$_{AMPPNP}$ cryo-EM map at overall

3.8 Å resolution. DeepEMhancer map contoured at 7σ. NTD1s are poorly resolved due to flexibility. **C**, As **B** but map surface coloured by local resolution. **D**, Fit of $CpaF_{AMPPNP}$ model in the associated map showcasing side chain detail. Zoom box shows map of NTD1 from a masked focussed refinement (Supplementary Figure 2B and 2C). Resolution was sufficient for an Alphafold model of the NTD1 3-helix motif to be rigid body fitted and modelled with ISOLDE. **E,** EM processing workflow summary.

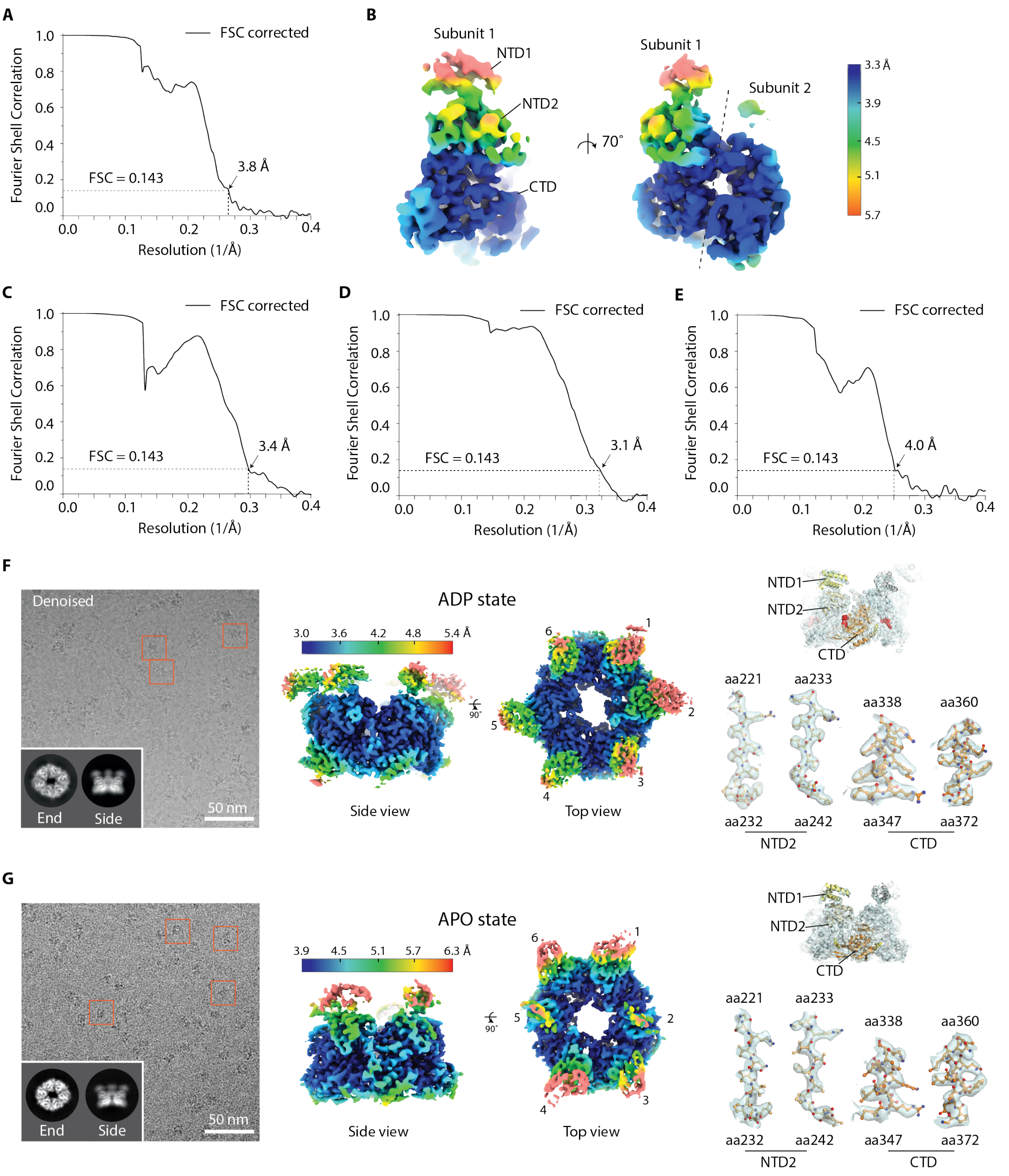

Supplementary Figure 2. Gold standard FSC curves and CpaF$_{ADP}$ and CpaF$_{APO}$ cryo-EM maps and model fit. **A**, FSC curve for CpaF$_{AMPPNP}$ map. **B**, Masked focussed refinement of the CpaF$_{AMPPNP}$ map zoned on subunit 1. The neighbouring subunit 2 is partially resolved despite being masked out. The map was surface coloured by local resolution, low-pass filtered at 4.5 Å for structure fitting and modelling with contour at 13σ. **C**, FSC curve for the masked focussed refinement. **D**, FSC curve for CpaF$_{ADP}$ map. **E**, FSC curve for CpaF$_{APO}$ map. **F**, (Left) CpaF incubated with ADP showing a typical cryo-EM micrograph with associated class

averages (inset). Micrograph was denoised using Topaz Denoise. (Middle) DeepEMhancer $CpaF_{ADP}$ map at 3.1 Å resolution overall coloured by local resolution and contoured at 3σ. (Right) Fit of $CpaF_{ADP}$ model in the associated map showcasing side chain detail. **G**, (Left) CpaF in the absence of nucleotide (APO) showing a typical cryo-EM micrograph with associated class averages (inset). (Middle) DeepEMhancer $CpaF_{APO}$ map at 4.0 Å resolution overall coloured by local resolution and contoured at 3σ. (Right) Fit of $CpaF_{APO}$ model in the associated map showcasing side chain detail contoured between 3-4σ.

```
                                    NTD0
                                                                  H0
Cc      MFGKRDSSASGDPK-APPPAPAGGAAIATRPQRVEPVAAPEPKAPAPKVSGPAPKPTVGL 59
Aa      ------------------------------------------------------------ 0
At      MFGKRGPEGPARSTKLEPVAPAVSMQ---------VATASEPRSPATDTLEPGQSPVTR- 50
Ms      ------------------------------------------------------------ 0
Mt      ------------------------------------------------------------ 0
Pa      -----------------------------------------------MSTGFGAR----- 8
Hd      ------------------------------------------------------------ 0

                NTD0                            NTD1
          H0                        H1                         H2
Cc      EQLRAAQGQPQTANIVREQSDYYHATKTTIFNALLNTIDLSQLAQLDLKQA--GEEIRDI 117
Aa      --------------MLT------KQQKILLRSEVLSNLDIEKIDELQSERSSLVNELVQI 40
At      ---QVTTGAPLSQKK-RARTEDYYNTKSQVFSALIDTIDLSQLAKLDAESA--REEIRDI 104
Ms      -----------------------------MSPSLIDRVRERLV----AETAPLSPDVVA- 26
Mt      ------------------------------------------------------------ 0
Pa      ---------PDGGAFHSRQEQDIQALKLRLHRYIIDEIDEDGMNLLEGARSAVVQYVSEK 59
Hd      --------------MLT------KDQQVFFRNALLSNLNVDTLDEIENERSKLVTELTQS 40

                      NTD1                                NTD2
          H2                   H3                H4       S1         S2
Cc      VAELVAIKNVSMSVAEQEHLVQDIINDVLGYGPLEPLLARDDIADIMVNGAHRVFIEVGG 177
Aa      VNRVANKSGAYLTSADTLVMAEIVADEIEGYGPLRDLMADDTINDILVNGPNDIWVERAG 100
At      VNDIITIKNFAMSIAEQEELLEDICNDVLGYGPLEPLLARDDIADIMVNGSGQTFIEVGG 164
Ms      -AAIRAESGGLLGDTEVLSNLRALDTELTGAGVLEPLLRADGTTDVLVTAPDAVWVDAGH 85
Mt      ----------MLGDTEVLANLRVLQTELTGAGILEPLLSADGTTDVLVTAPDSVWVDDGN 50
Pa      VCEYGSRHQLAISRYELDRLAEEVVDELTGFGPLEILLRDPGVSEILVNGPGRVFVEREG 119
Hd      LYRVANTNNIYITPYDATDMAEIVADEIGGYGPIRELMEDDTVNDILVNGPDNIWIERAG 100
                   :   :     . :  :: * * :. *:      :::*..    :::

                                       NTD2
        S3     S3'         H5                      S4             S5
Cc      KVQLTNVRFRDNLQLMNICQRIVSQVGRRVDESSPICDARLPD------GSRVNVIAPPL 231
Aa      ILEKTDKEFVSNEQLTDIAKRLVARVGRRIDDGSPLVDSRLPD------GSRLNVVIAPI 154
At      KTIESDIRFRDNAQLLSICQRIVSQVGRRVDESSPICDARLPD------GSRVNVIAPPL 218
Ms      GLRRTDIRFPDEAAVRRLAQRLALLAGRRLDEAQPWVDGHLGGM--GPFTVRLHAVLPPI 143
Mt      GLRRSQIRFADESAVRRLAQRLALAAGRRLDDAQPWVDGQLTGIGVGGFAVRLHAVLPPV 110
Pa      RLYQSDLRFIDDHHVLRVIQRILAPLGRRLDESSPMVDARLPD------GSRVNAIIPPV 173
Hd      VLEKTNKTFINNEQLTDIAKRLVARVGRRIDEGMPLVDSRLPD------GSRLNVVIQPI 154
            ::  * .:  :  : :*:    ***:*:. *  *.:* .        *::.:  *:

          NTD2                              CTD          Walker A/P-loop
             S6                H6             H7              S7      H8
Cc      ALDGPTLTIRKFKKDKLTMKNLVEFASISPEGARVLGVIGACRCNLVISGGTGSGKTTLL 291
Aa      ALDGTSISIRKFSKNKKTLQELVNFGSMTREMANFLIIAARSRVNIIVSGGTGSGKTTLL 214
At      AIDGPALTIRKFKRDKLTLDQLVRFGAITPEGATLLKIIGRVRCNVVISGGTGSGKTTLL 278
Ms      AAAGTCLSLRVLRPATQNLDSLARSGAIAPAAVDLLRRVIDARLAFLISGGTGAGKTTLL 203
Mt      ATQGTCLSLRVLRPATQDLAALAAAGAIDPAAAALVADIVTARLAFLVCGGTGAGKTTLL 170
Pa      ALDGPCISIRKFSQELLRSADLLAYQSVDEALLEFLRQAVSRRCNILISGGTGTGKTTLL 233
Hd      ALDGTSISIRKFSKSKKSLQELVNFGSMTLDMANFLIIAARSRVNIIVSGGTGSGKTTLL 214
        *  *  :::* :         *    ::      .:      *  .::.****:******

                 CTD      ASP box                CL
         H8             S8              S9                    H9
Cc      NTMTAFIDPTERVVTCEDAAELQLQQPHVVRLETRPPNLEGSGAVTMRDLVKNCLRMRPE 351
Aa      NALSNYISHTERVITLEDTAELRLEQPHVVRLETRLAGVEHTGEVTMQDLVINALRMRPE 274
At      NCLTSFIDKDERVITCEDTAELQLQQPHVVRLETRPPNIEGEGEITMRDLVKNCLRMRPE 338
Ms      SALLGTVPGHERIVCVEDAAELAPQHPHVVRLVARNANVEGVGEITVRDLVRQALRMRPD 263
Mt      AAMLGAVSPDERIVCVEDAAELAPRHPHLVKLVARRANVEGIGEVTVRQLVRQALRMRPD 230
Pa      NVISGFIDERERIVTIEDTAELQLGHDHVVRLETRPPNAEGYGEVTARDLIRNALRMRPD 293
Hd      NALSSYISPTERVLTLEDTAELRLEQPHVVRLETRLAGVERTGEITMQDLVINALRMRPE 274
          :   :   **::  **:***   : *:*:* :*  . *  * :* ::*: :.*****:
```

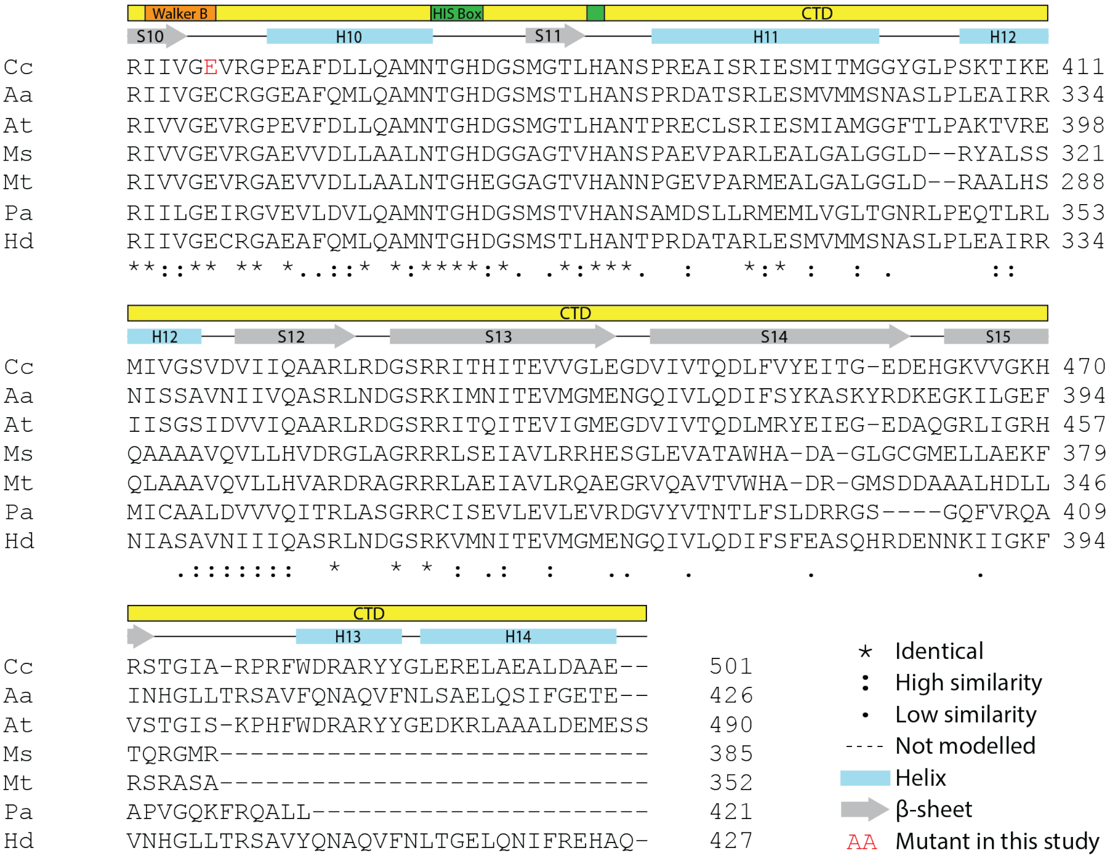

Supplementary Figure 3. CpaF sequence alignments and secondary structure assignment. Sequences from *Caulobacter crescentus* (Uniprot code A0A0H3CDS2), *Aggregatibacter actinomycetemcomitans* (Uniprot code E1CIZ1), *Agrobacterium tumefaciens* (Uniprot code A0A083ZNK9), *Mycobacterium smegmatis* (Uniprot code I7FU99), *Mycobacterium tuberculosis* (Uniprot code A0A0H3LFC8), *Pseudomonas aeruginosa* (Uniprot code Q9HW98) and *Haemophilus ducreyi* (Uniprot code Q8KQI7).

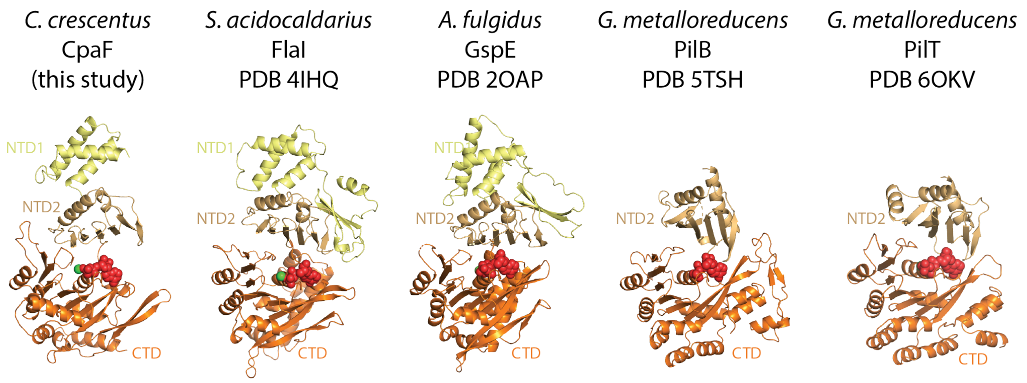

Supplementary Figure 4. Gallery of CpaF homologues. CpaF shares closest structural homology with the archaeal proteins FlaI and GspE. PilB and PilT are unidirectional motors dedicated to pilus extension and retraction, respectively. Nucleotides shown as red spheres, Mg^{2+} as green sphere.

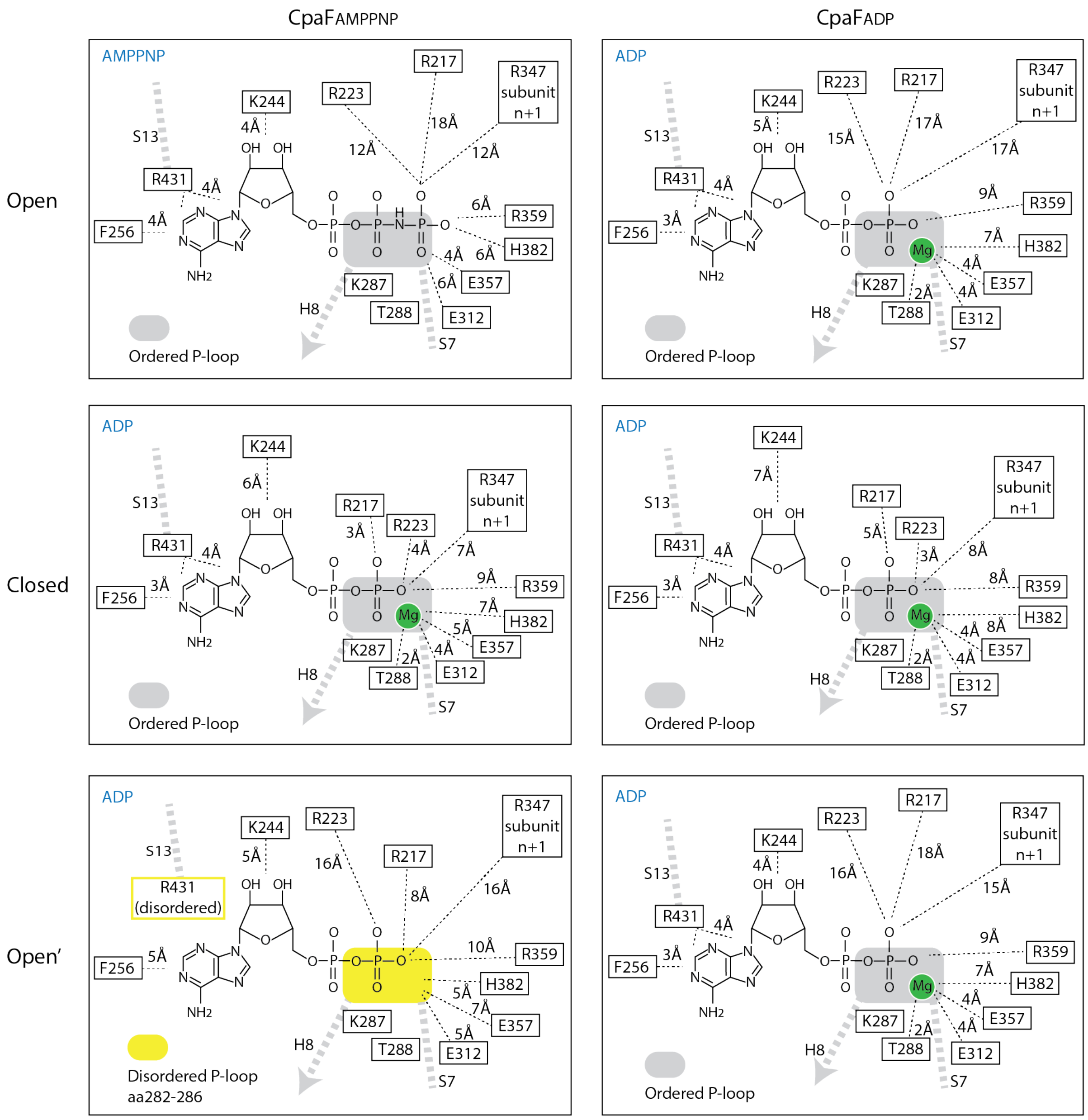

Supplementary Figure 5. Schematics of $CpaF_{AMPPNP}$ and $CpaF_{ADP}$ nucleotide binding pockets with key nucleotide interfacing residues shown. K287 and T288 form part of the P-loop consensus sequence. E312, E357 and H382 constitute part of the ASP box, Walker B motif, and HIS box, respectively. Note that K244R (in combination with F243L) and D309N (forms part of the ASP box with E312) mutants exhibit reduced pilus extension and retraction rates[26]. The distance between the Mg^{2+} and the P^{γ} proximal oxygen is 1.8 Å for all states except for $CpaF_{ADP}$ open' state where the distance is 2.2 Å.

```
                                           NTD0

CpaF_Cc      MFGK-------R-D---SS---------------------------------------AS 10
GspE_2OAP    MAKN-------HYDILRRH---------------------------------------IR 14
FlaI_4II7    -MSF-------VEDYLTKL---------------------------------------QE 13
PilT_6OKV    ------------------------------------------------------------ 0
GspE_4KSR    ------------------------------------------------------------ 0
PilB_5TSH    MQASRLGELLVRNNVITKEQLAKALEEQKSADGQQRLGSILIKNGLISEPDLTSFLSKQY 60

                                           NTD0
                                                                     H0
CpaF_Cc      GDPKAPPPAPAGGAAIA-------TRPQRVEPVAAPEPKAPAPKVSGPAPKPTVGLEQLR 63
GspE_2OAP    SEDLLETPEFGSGSRIV-------EEYWIQEPFTKA------------------II--VE 47
FlaI_4II7    RPTIIENPNILKGSKIF-------NAIYRVDDFVYI------------------HIQSIK 48
PilT_6OKV    ------------------------------------------------------------ 0
GspE_4KSR    ------------------------------------------------------------ 0
PilB_5TSH    GVPSINLSEFEAEQAVVKIIPADVAQKYQIVPVNRAG---STLIIAMADPSNIFAIDDIK 117

                       NTD0                          NTD1
              H0                                H1
CpaF_Cc      AAQGQPQTANIV------REQSDYYHATK-------TTIFNALLN---TIDLSQLAQL-- 105
GspE_2OAP    NEDEF---------------RNVYYALEPTVS-SEEAEVISALYDDLKKILVLQDVSV-- 89
FlaI_4II7    SEDGY----------------NQYNVIEPPRPTHDEMEEIEEKFA---LSIGDKEPPE-- 87
PilT_6OKV    ------------------------------------------------------------ 0
GspE_4KSR    -------------------------------------------MDFFSLA--EELPQNED 15
PilB_5TSH    FMTGYNVEVVVASESAIKAAIDKYYDQSASLA--DVMGDL--EMDDLEVIDTDDEVDVSS 173

                                 NTD1                                  NTD2
                            H2                     H3                H4
CpaF_Cc      -----DLKQAGEEIRDIVAELVAIKNVSMSVAEQEHLVQDIINDVLGYGPLEPLLARDDI 160
GspE_2OAP    -----DLEERAEVLVRAIEKLSKEYAVSFTDNFYSRMLYYLFRDFFGYGLIDPLMEDTNV 144
FlaI_4II7    -----DTKEKEKLIRSILDKILLRMRLSVPK---EYVIYHFIRDKLYTGSLEPLIRDPYI 139
PilT_6OKV    ----------MANMHQLLTELVN----------------------------------RGG 16
GspE_4KSR    LLESEDDAPIIKLINAMLGEAIK----------------------------------EGA 41
PilB_5TSH    LERATEDAPVVKLVNLILTDAIK----------------------------------RKA 199
                          :   : .                          * *

                                          NTD2
               S1         S2      S3     S3′         H5                  S4
CpaF_Cc      ADIMVNGAHR-VFIEV--GGKVQLTNVRFRDNLQLMNICQRIVS-QVGRRVDESSPICDA 216
GspE_2OAP    EDISCDGYNIPIFIYHQKYGN-VETNIVLD-QEKLDRMVLRLTQ-RSGKHISIANPIVDA 201
FlaI_4II7    EDISIPGLGH-VYIVHKVFGP-MRTSIKFENYEELDNLIVSLSE-KSYRPVSHNRPVVDA 196
PilT_6OKV    SDLHLTTNS---PPQIRIDGKLLPLDMPPLN---AVDTKQLCYS------ILTEQ--QKH 62
GspE_4KSR    SDIHIETFEKTLSIRFRVDGVLREVLAPSRK---LSSLLVSRVK------VMAKLDIAEK 92
PilB_5TSH    SDIHIEPYERSFRVRYRIDGVLYEVMKPPLK---LKNAITSRIK------IMAELDIAER 250
              *:                *                           **  :

                               NTD2                                 CTD
                    S5                              S6             H6
CpaF_Cc      RLPDGSRVNVI-APPL--------------ALDGPTLTIRKFKKDKLTMKNL--VEFASI 259
GspE_2OAP    TLPDGSRLQATFGTEV--------------TPRGSSFTIRKFTIEPLTPIDL--IEKGTV 245
FlaI_4II7    SLPDGSRVNFVYGVDI--------------SRRGSNLTVRKFSRVPTSITQL--IMFGTL 240
PilT_6OKV    KFEENNELDLSFGIKGLSRFRGNVFV----QRGAVAGVFRVIP---Y--KILSFEELG-L 112
GspE_4KSR    RVPQDGRISLRIGG-RAVDVRVSTMP----SSHGERVVMRLLD---KNATRLDLHSLG-M 143
PilB_5TSH    RLPQDGRIKIKLGGGQDMDYRVSVLP----TLFGEKVVLRLLD---KSNLQLDMTKLG-Y 302
                   . .                        .   ..* :         *
```

```
           CTD  Walker A/P-loop  CTD  ASP box
           H7  S7  H8  S8  H8'
CpaF_Cc    SPEGARV-LGVIGACRCNLVISGGTGSGKTTLLNTMTAFIDP----TERVVTCEDAAELQ 314
GspE_2OAP  PSGVLAY-LWLAIEHKFSAIVVGETASGKTTTLNAIMMFIPP----DAKVVSIEDTREIK 300
FlaI_4II7  SSMMAAY-IWTMLDEGMNLFVCGETASGKTTTLNAITAFIPP----NLKIVTIEDTPELT 295
PilT_6OKV  PP----VVRELAEKPRGLVLVTGPTGSGKSTTLAAIIDKINTD--RHEHIVTVEDPIEYL 166
GspE_4KSR  TAHNHDNFRRLIKRPHGIILVTGPTGSGKSTTLYAGLQELNSS---ERNILTVEDPIEFD 200
PilB_5TSH  EPDALHYFKEAIHKPFGMVLVTGPTGSGKTVSLYSALGELNKT---TENISTAEDPVEFN 359
                              .: * *.***:. * :    :        .: : *.  *

           CTD  CL  CTD  Walker B  HIS Box
           S9  H9  S10  H10
CpaF_Cc    LQQPHVVRLETRPPNLEGSGAVTMRDLVKNCLRMRPERIIVGEVRGPEA-FDLLQAMNTG 373
GspE_2OAP  LYHENWIAEVTR--TGMGEGEIDMYDLLRAALRQRPDYIIVGEVRGREA-QTLFQAMSTG 357
FlaI_4II7  VPHSNWVAEVTR--ETGGEGTIKLFDLLKAALRQRPNYILVGEIRDKEG-NVAFQAMQTG 352
PilT_6OKV  HPHK---SCVVNQR-EVGADTKSFKNALKYILRQDPDVVLVGELRDLETIEAALTLAETG 222
GspE_4KSR  IDGI---GQTQVNP-RV---DMTFARGLRAILRQDPDVVMVGEIRDLETAQIAVQASLTG 253
PilB_5TSH  FAGI---NQVQMHE-DI---GLNFAAALRSFLRQDPDIIMIGEIRDFETAEIAIKAALTG 412
                            * *   :   ::  **  *  :::** *. *     .    **

           CTD
           S11  H11  H12  S12  S13
CpaF_Cc    HDGSMGTLHANSPREAISRIESMITMGGYGLPSKTIKEMIVGSVDVIIQAARLRDGSRRI 433
GspE_2OAP  HA-SYSTLHAGDINQMVYRLESE--------PLKVPRSMLQFLDIALVQTMW-VRGNTRL 407
FlaI_4II7  HS-VMATFHAANITTLIQRLTGY--------PIEVPKSYINNLNIALFQTALYDKKGNLI 403
PilT_6OKV  HL-CFATLHTNSAVQTINRIVDVFPSY--QQP-QVRAQLSFVLEGVLSQTLLPKASGTGR 278
GspE_4KSR  HL-VMSTLHTNTAVGAVTRLRDMGIEP--F---LI----SSSLLGVLAQRLVRTL----- 298
PilB_5TSH  HL-VLSTLHTNDAPATINRLLNMGVEP--F---LV----ASAVNLITAQRLARRV----- 457
           *     *:*:      :  *:                            *

           CTD
           S13  S14
CpaF_Cc    THITEV---VGL---------EGDVIVTQDLF------------VYEITGE-----DEHG 464
GspE_2OAP  RRTKEV---NEI---------LGIDPVDKNLLVN-------QFVKWDPKED------KH- 441
FlaI_4II7  RRVVEV---DEI---------IDIDPVTNDVVYI-------PAFTYDSVQD------KM- 437
PilT_6OKV  VLAIEVMVPNPAI-----RNLIREDKIHQIYSQMQVGQEKFGMMTMNQCLYGLLQKRHIT 333
GspE_4KSR  --CPDCKEPYEADKEQR--KLFDSKKKEPLILYRA-----TGCPKCNHKGYR---G---- 342
PilB_5TSH  --CSECKQPEEIPIQALIDAGVSPDEGPSYVCYKG-----TGCVKCNNTGYK---G---- 503
               :                                         :

           CTD
           S15  H13  H14
CpaF_Cc    KVVGKH-----------------RSTGIARPRFWDRARYYGLER--ELAEALDAAE---- 501
GspE_2OAP  IEVSM------PKKLEKMADFLGVSVQEVYDEMLSRKRYLELMLKRGIRNYKEVTR-YIH 494
FlaI_4II7  LFAGKGSSYLIENKIAVKRGIDRRNIGLLYDELQMRSRFLNLLVEKKIFNYYDVWD-YIL 496
PilT_6OKV  MDVGMG-RSPDPD------------------------ELKQMLTS-GVRPQAP-R----- 361
GspE_4KSR  -RTGIHELLLVDD------------------------ALQELIHS-EAGEQAM-EKHIRA 375
PilB_5TSH  -RVGFYQVMPMLE------------------------EIRELILN-GANTAEIKRESMRL 537
              .                                     :

           CTD
CpaF_Cc    -------------------------------       501
GspE_2OAP  AYYRNPELAMTKMEEGL--------------       511
FlaI_4II7  RARQM------GLEEAIKYVSNI--------       513
PilT_6OKV  --PPMR-------------------------       365
GspE_4KSR  TTPSIRDDGLDKVRQGITSLEEVMRVT----       402
PilB_5TSH  GIKTMRQSGLTKLKEGVTSFEEVLRVTVADD       568
```

- * Identical
- : High similarity
- . Low similarity
- ---- Not modelled
- Helix
- β-sheet
- AA mutant in this study
- */A Identical within the TadA/archaeal GspE/FlaI clade

Supplementary Figure 6. Sequence alignments of selected type 4 filament family CpaF homologues. Sequences from *Caulobacter crescentus* CpaF (Uniprot code A0A0H3CDS2),

Archaeoglobus fulgidus GspE2 (Uniprot code O29598), *Sulfolobus acidocaldarius* FlaI (Uniprot code Q4J9L0), *Geobacter metallireducens* PilT (Uniprot code Q39VU6), *Vibrio cholerae* GspE (Uniprot code P37093) and *Geobacter metallireducens* PilB (Uniprot code Q39VU7).

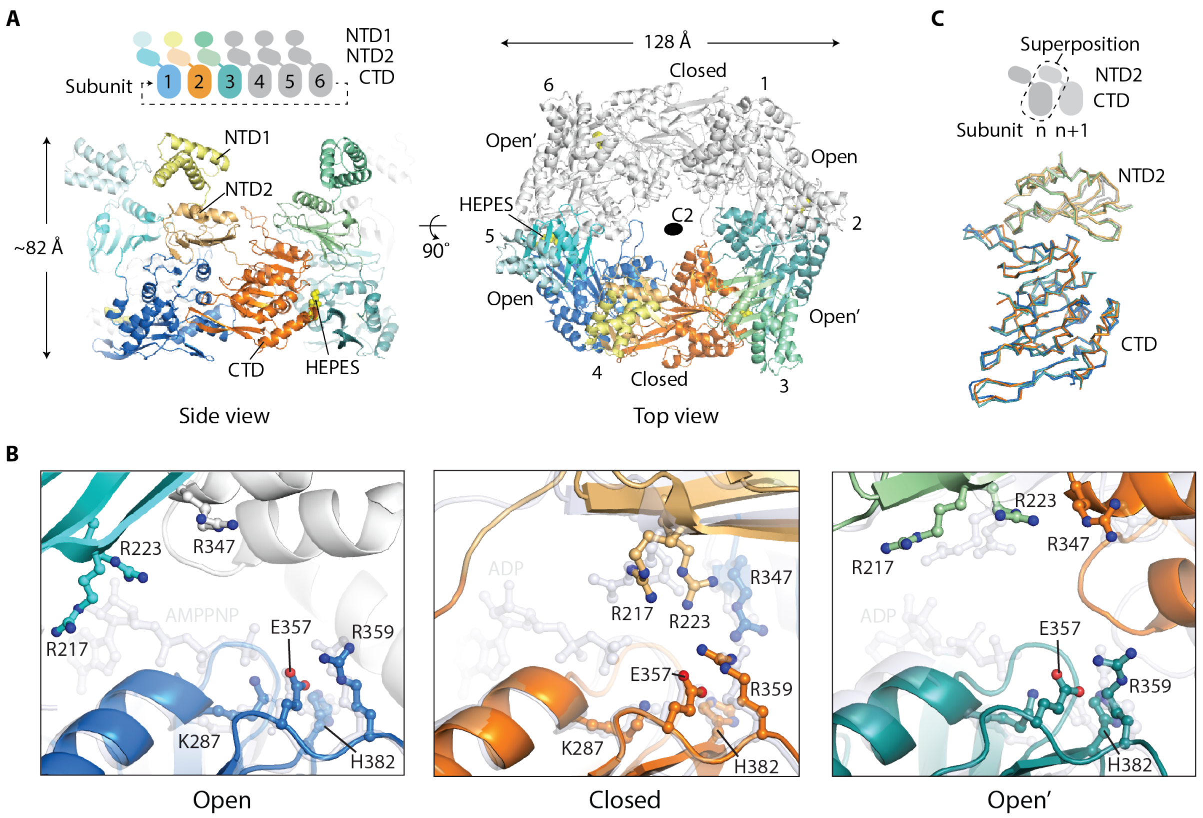

Supplementary Figure 7. Cryo-EM structure of CpaF$_{APO}$. A, Model of the C2 symmetric CpaF hexamer with no ligand observed in the nucleotide binding pockets. HEPES was bound in the CTD/CTD$_{n+1}$ clefts in the open and open' states. **B**, Analysis of CpaF$_{APO}$ open, closed and open' active site states. To aid comparison, active sites from CpaF$_{AMPPNP}$ have been superposed and coloured grey with 50 % transparency. For the open state active site, only the CpaF$_{AMPPNP}$ CTD is superposed for comparison as in the absence of nucleotide the CpaF$_{APO}$ NTD2 is rotated relative to the CpaF$_{AMPPNP}$ NTD2 and does not align closely. No Mg^{2+} was observed in the nucleotide binding pockets. **C**, Superposition of the three unique NTD2/CTD$_{n+1}$ units within a CpaF hexamer showing close similarity. For each subunit, the NTD2 of subunit n interfaces with the CTD of subunit n+1 in a domain swap.

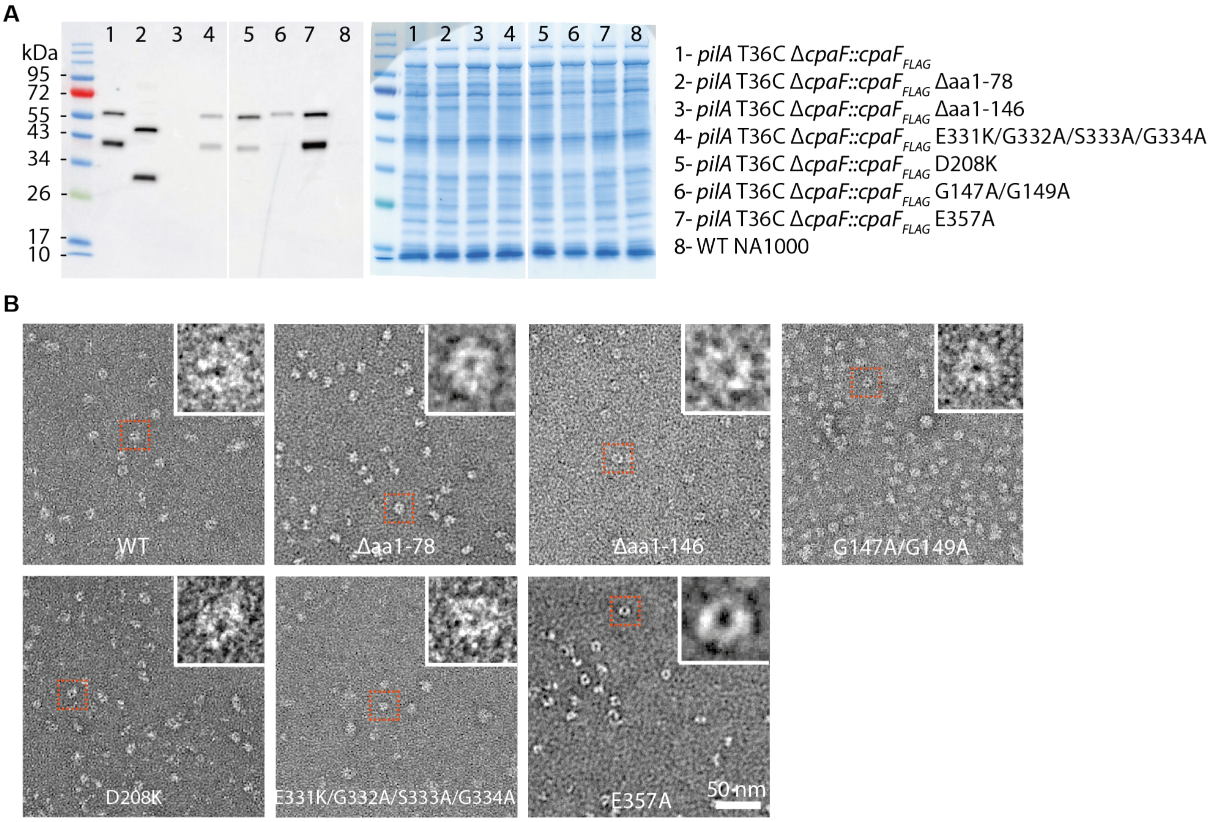

Supplementary Figure 8. CpaF mutant *in vivo* and *in vitro* expression with fluorescent quantification. A, (Left) *C. crescentus* cell lysate anti-FLAG Western blot detecting CpaF expression in wildtype and truncated or mutant strains. All complemented CpaF constructs incorporate a C-terminal FLAG tag. Expression was detected for all CpaF variants except CpaFΔaa1-146. (Right) SDS-PAGE gel showing equivalent quantities of loaded samples used for the Western blot shown in the left panel. Source data are provided as a Source Data file. **B**, *In vitro* expression in *E. coli* of the CpaF truncations or mutant strains showing hexamer assembly in all cases.

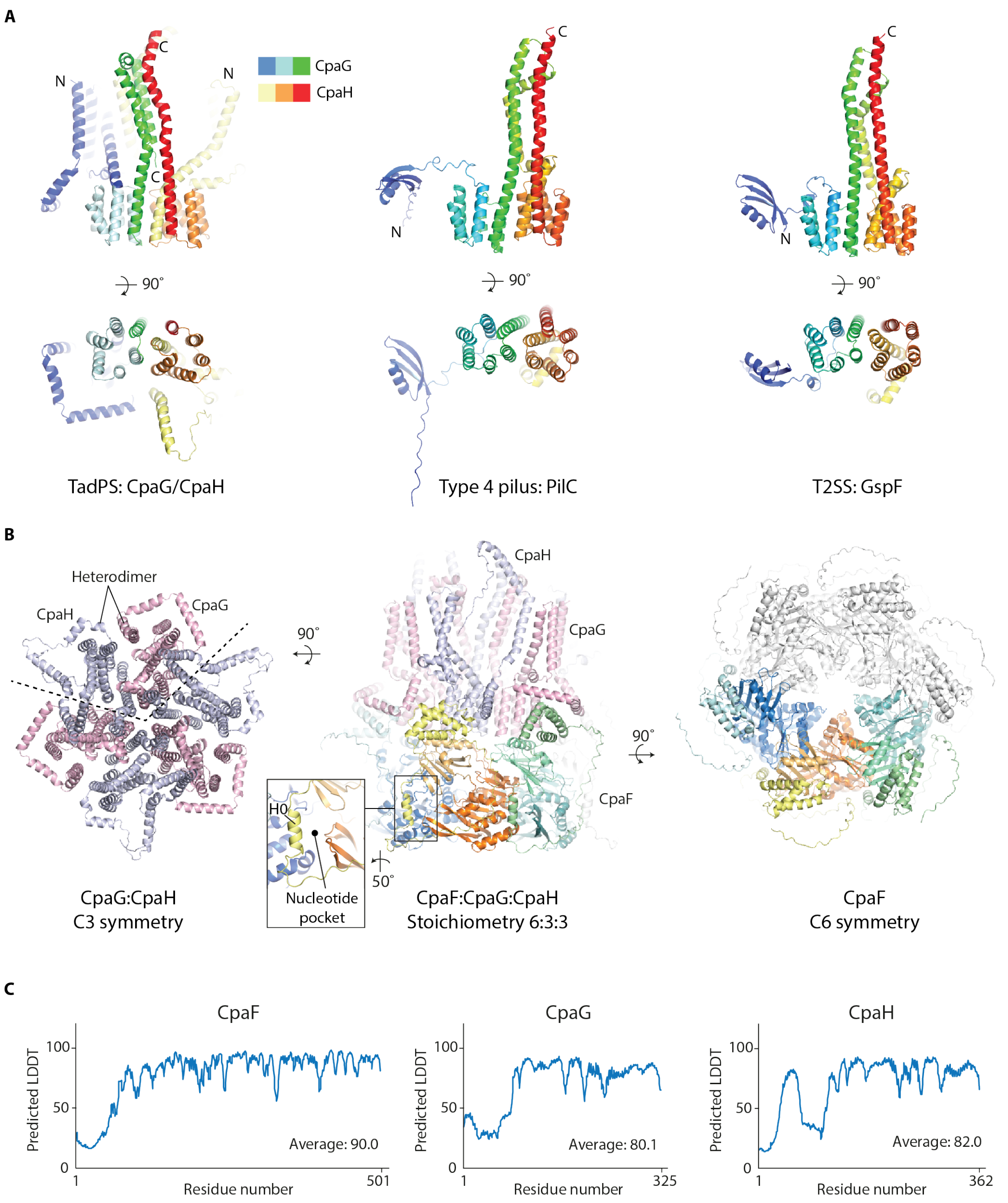

Supplementary Figure 9. *In-silico* modelling of CpaF with CpaG/CpaH, relating to Figure 5. **A**, Alphafold models CpaG and CpaH as a heterodimer with a similar fold as PilB (Type 4 pilus system) and GspF (T2SS). Rainbow colouring with blue N-terminus through to red C-terminus with CpaG and CpaH coloured as a continual chain so as to highlight the common fold with PilB and GspF. **B**, (Centre panel) Alphafold model with three CpaG and CpaH subunits bound to six CpaF subunits. Each CpaG and CpaH pair form the equivalent heterodimer as shown in **A**. This same model is shown in Figure 5A with two CpaG and CpaH heterodimers omitted for clarity. (Left panel) Three CpaG and CpaH heterodimers model with C3 symmetry. CpaF has been omitted for clarity. (Right panel) CpaF models as a C6 hexamer and each subunit has an

RMSD Cα = 2.2 Å relative to the experimentally determined $CpaF_{AMPPNP}$ closed state subunit. CpaG and CpaH have been omitted for clarity. **C**, Alphafold per residue model confidence scores (pLDDT) for CpaF, CpaG and CpaH relating to the model shown in **B** and Figure 5A. Overall pLDDT score = 74.9.

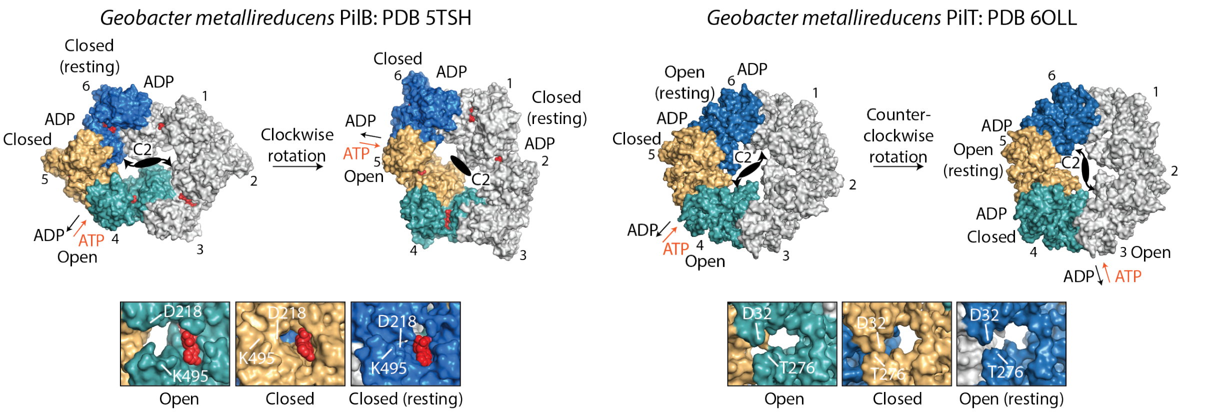

Supplementary Figure 10. Nucleotide cycling in PilB[27] and PilT[33] with associated subunit state. (Left) PilB forms a C2 symmetric hexamer with subunits in open, closed and closed (resting) states. Zoom panels show side view of the nucleotide binding pockets. The open state enables nucleotide exchange with ATP binding, the closed state promotes ATP hydrolysis, whilst the closed resting state retains ADP that is primed for release in the subsequent open step. This sequence of nucleotide cycling and associated conformational change induces the C2 symmetry axis to rotate in a clockwise motion. (Right) PilT forms a C2 symmetric hexamer with subunits in open, closed and open (resting) states. Nucleotide cycling is proposed to support C2 symmetry axis rotation in a counter-clockwise motion. This PilT structure was determined in the absence of nucleotide likely resulting in relatively 'relaxed' open, closed and open (resting) states. PilT from *Aquifex aeolicus* crystallised in the presence of ADP also shows C2 symmetry but with accentuated open, closed and open (resting) states that may better represent the *in vivo* conformation when nucleotide is present[32].

Supplementary Table 1. Cryo-EM data collection, refinement and validation statistics

**Data collection**	**AMPPNP dataset**	**ADP dataset**	**Apo dataset**
Electron microscope	Titan Krios	Titan Krios	Titan Krios
Voltage (kV)	300	300	300
Pixel size (Å)	1.1	0.829	0.85
Electron exposure ($e^-/Å^2$)	40	40	40
Defocus range (μm)	0.8-2.8	0.5-2.1	0.5-2.5
Images	5,108	24,809	21,498
**3D reconstruction**			
Final particles	73,200	114,641	51,697
Resolution (Å)	3.8	3.1	4.0
FSC threshold	0.143	0.143	0.143
B factor ($Å^2$)	118	84	80
**Refinement**			
Model	CpaF aa80-503	CpaF aa80-502	CpaF aa80-502
Chains	12	12	10
Ligands	12	16	4
**Model Composition**			
Total atoms	36340 (H: 17724)	36930 (H: 18102)	36618 (H: 18006)
Total residues	2514	2544	2538
**R.m.s. deviations**			
Bond length (Å)	0.002	0.004	0.005
Angles (°)	0.593	0.567	0.622
**Validation**			
MolProbity score	2.03	1.78	2.42
Clashscore	8.33	6.05	10.41
Poor rotamer (%)	1.81	1.41	2.61
**Ramachandran plot (%)**			
Outliers	0	0	0
Allowed	5.57	4.82	10.77
Favoured	94.43	95.18	89.23
**B-factors/ADP**			
Minimum	30.00	30.00	30.00
Maximum	120.48	152.66	301.22
Mean	77.02	71.71	133.22
**Model vs. Data**			
CC (mask)	0.70	0.82	0.68
CC (volume)	0.69	0.81	0.69

Supplementary Table 2. Primers

Primer names	Sequences (5' -> 3')
**FX cloning**	
cpaF WT	
CpaF_for	atatatgctcttctagttttggaaagcgcgactcgtcagcctcc
CpaF_rev	tatatagctcttcatgcttccgcagcgtcgagggcttcggccag
cpaF truncations	
CpaF_Δaa1-78_for	atatatgctcttctagtagtgactactaccacgccaccaagacc
CpaF_Δaa1-78_rev	tatatagctcttcatgcttccgcagcgtcgagggcttcggccag
CpaF_Δaa1-146_for	atatatgctcttctagtggttatggtccgctggagccgctgctg
CpaF_Δaa1-146_rev	tatatagctcttcatgcttccgcagcgtcgagggcttcggccag
**Gibson cloning**	
cpaF mutants	
CpaF_G147A/G149A_for	ccgctggagccgctgctggc
CpaF_G147A/G149A_rev	cggctccagcggggcataggcgaggacgtcgttgatgatgtcctggaccagatgc
CpaF_D208K_for	gaaagctcgccgatctgcgatgcgcg
CpaF_D208K_rev	agatcggcgagctttccttgacccggcggccgacctgg
CpaF_E331K/G332A/S333A/G334A_for	gcggtgaccatgcgggatctggtca
CpaF_E331K/G332A/S333A/G334A_rev	cgcatggtcaccgcggctgcggccttgagattgggcggacgggtttccaggc
CpaF_E357A_for	gtccgtggccccgaggcgt
CpaF_E357A_rev	ggggccacggactgcgccgacgatgatccgctcgggacg
***C. crescentus* mutants**	
cpaF deletion	
NTToligo4020	gcaattgaagccggctggcgccaacgtcggcgatgctggggtcgc
NTToligo4021	ttccaggcccacggtcggcttgg
NTToligo4022	ccaagccgaccgtgggcctggaagaaggcgacgtgatcgtcaccc
NTToligo4023	cggccgaagctagcgaattcgtgccagaacatcttgacgttgtcgc
pilA deletion	
NTToligo2978	gcaattgaagccggctggcgccaacgtcggcgatgctggggtcgc
NTToligo2979	cttggtcatgacttggtctcctaaac
NTToligo2980	taggagaccaagtcatgaccaagggcacctaagccactcgcgtcacg
NTToligo2981	cggccgaagctagcgaattcgtgcccgccaggccggtgtacagcatg
pilA mutation (T36C)	
NTToligo4018	gcagacggcggtcacgatgacaacg
NTToligo4019	cgttgtcatcgtgaccgccgtctgcaccctcggcaccaacctgcgcac

Supplementary Table 3. Plasmids and strains

**Plasmid name**	**Purpose**	**Origin**
pBXC3H	Recombinant expression vector *E. coli*	Lab collection
pBXC3H_*cpaF*	Expression of WT CpaF	This work
pBXC3H_*cpaF*_G147A/G149A	Expression of mutant CpaF	This work
pBXC3H_*cpaF*_D208K	Expression of mutant CpaF	This work
pBXC3H_*cpaF*_E331K/G332A/S333A/G334A	Expression of mutant CpaF	This work
pBXC3H_*cpaF*_E357A	Expression of mutant CpaF	This work
pBXC3H_*cpaF*_Δaa1-78	Expression of mutant CpaF	This work
pBXC3H_*cpaF*_Δaa1-146	Expression of mutant CpaF	This work
pNPTS138	Integrative vector for *Caulobacter* gene knockout/knock-in	Lab collection
pXYFPC-1	Integrative vector for gene insertion at the *xylX* locus	Lab collection
pNPTS138::Δ*cpaF*	Knockout plasmid *cpaF*	This work
pNPTS138::Δ*pilA*	Knockout plasmid *pilA*	This work
pNPTS138::*pilA* (T36C)	Knock-in plasmid *pilA* (T36C)	This work
pXYFPC-1::*cpaF*-FLAG	Knock-in plasmid *cpaF*	This work
pXYFPC-1::*cpaF* (Δaa1-78)-FLAG	Knock-in plasmid *cpaF* (Δaa1-78)	This work
pXYFPC-1::*cpaF* (Δaa1-146)-FLAG	Knock-in plasmid *cpaF* (Δaa1-146)	This work
pXYFPC-1::*cpaF* (G147A/G149A)-FLAG	Knock-in plasmid *cpaF* (G147A/G149A)	This work
pXYFPC-1::*cpaF* (D208K)-FLAG	Knock-in plasmid *cpaF* (D208K)	This work
pXYFPC-1::*cpaF* (E331K/G332A/S333A/G334A)-FLAG	Knock-in plasmid *cpaF* (E331K/G332A/S333A/G334A)	This work
pXYFPC-1::*cpaF* (E357A)-FLAG	Knock-in plasmid *cpaF* (E357A)	This work

**Strain name**	**Purpose**	**Origin**
E. coli MC1061	CpaF expression strain	Lab collection
Caulobacter crescentus NA1000	Wildtype synchronisable *C. crescentus* strain	Lab collection
NA1000 Δ*pilA*	*pilA* knockout strain	This work
NA1000 *pilA* (T36C)	PilA pilus Alexa labelling strain	This work
NA1000 *pilA* (T36C) Δ*cpaF*	*cpaF* knockout strain	This work
NA1000 *pilA* (T36C) Δ*cpaF*::pXYFPC-1-*cpaF*-FLAG	Complementation control strain	This work
NA1000 *pilA* (T36C) Δ*cpaF*::pXYFPC-1-*cpaF* (Δaa1-78)-FLAG	Complementation strain	This work
NA1000 *pilA* (T36C) Δ*cpaF*::pXYFPC-1-*cpaF* (Δaa1-146)-FLAG	Complementation strain	This work
NA1000 *pilA* (T36C) Δ*cpaF*::pXYFPC-1-*cpaF* (G147A/G149A)-FLAG	Complementation strain	This work
NA1000 *pilA* (T36C) Δ*cpaF*::pXYFPC-1-*cpaF* (D208K)-FLAG	Complementation strain	This work
NA1000 *pilA* (T36C) Δ*cpaF*::pXYFPC-1-*cpaF* (E331K/G332A/S333A/G334A)-FLAG	Complementation strain	This work
NA1000 *pilA* (T36C) Δ*cpaF*::pXYFPC-1-*cpaF* (E357A)-FLAG	Complementation strain	This work
