# Supplementary material for: Bidirectional pilus processing in the Tad pilus system motor CpaF
Source: Nat Commun. 2024 Aug 5;15:6635. doi: 10.1038/s41467-024-50280-6 (PMC11300603; doi:10.1038/s41467-024-50280-6)
Supplement: Supplementary file 3 — Description of Additional Supplementary Files [file 41467_2024_50280_MOESM3_ESM.pdf]

Description of Additional Supplementary Files

File Name: Supplementary Movie 1
Description: Related to Figure 1C. Morph between subunits within the CpaFAMPPNP asymmetric unit.
